# A screening strategy for bioactive components from *Amaranth*: An integrated approach of network pharmacology, molecular docking and molecular dynamics simulation

**DOI:** 10.1371/journal.pone.0338443

**Published:** 2025-12-26

**Authors:** Zixuan Zhao, Zhengxin Huang, Kaiming Wang, Hua Zhang, Ying Ren, Jinjin Tong

**Affiliations:** 1 Animal Science and Technology College, Beijing University of Agriculture, Beijing, People's Republic of China; 2 STEM college, RMIT university, Melbourne, Victoria, Australia; 3 College of Veterinary Medicine, Beijing University of Agriculture, Beijing, People's Republic of China; 4 Hubei Key Laboratory of Animal Nutrition and Feed Science, Wuhan Polytechnic University, Wuhan, People's Republic of China; Universidad San Francisco de Quito - Campus Cumbaya: Universidad San Francisco de Quito, ECUADOR

## Abstract

*Amaranth* is a traditional medicinal and forage plant with promising anti-inflammatory properties. To enhance its utilization in livestock and feed industries, this study investigated the bioactive compounds and mechanisms of *Amaranth* at different growth stages using metabolomics and network pharmacology. LC-MS/MS identified 266 metabolites, including key compounds such as ferulic acid, isoferulic acid, sinapic acid, and 13-HODE. A total of 132 inflammation-related targets were screened, and enrichment analysis revealed their involvement in ATP binding, inflammatory response, and PI3K-Akt/MAPK signaling pathways. Molecular docking and molecular dynamics simulations confirmed strong interactions between core targets (e.g., *IL6*, *MMP9*) and major compounds. These findings demonstrate that phenolic acids and fatty acids in *Amaranth* possess anti-inflammatory activity, underpinning its prospective use in the formulation of biofunctional feeds and in promoting the health of livestock.

## Introduction

With the growing global challenge of food competition between humans and livestock, alongside increasing climate variability, forage shortages have become a critical issue in livestock production. The escalating demand for high-quality dairy products further intensifies the need for sustainable feed resources. Meta-analyses and recent projections indicate that total global food demand will rise by 35% to 60% between 2010 and 2050, depending on socioeconomic and climate scenarios [1]. As environmental pressures mount, the expansion of dairy farming to meet rising demand strains natural resources, exacerbating land degradation, water scarcity, and greenhouse gas emissions [2]. Ensuring adequate protein-rich feed is vital for maintaining milk production efficiency and quality, yet conventional feed sources often increase ecological burdens. Identifying and utilizing novel, sustainable feed alternatives are crucial for reducing dairy farming’s environmental impact and ensuring its long-term viability.

The growing demand for sustainable animal feed has driven research into alternative protein sources, including agricultural by-products, food industry residues, and underutilized crops [3]. Among these, *Amaranth* (*A. hypochondriacus*), once considered invasive weeds, are now recognized as pseudocereals with high nutritional and nutraceutical potential. Due to its high content of proteins, fats, and health-promoting phytochemicals such as flavonols, hydroxycinnamic acid derivatives, and benzoates, *Amaranthus* serves as a functionally beneficial feed component in animal diets. *Amaranth* thrives in poor soils and extreme climates, making it a promising forage resource for ruminants. The enrichment of oleic and linoleic acids in its seeds elevates the functional feed value of *Amaranthus* for ruminant animals [4]. Compared to corn silage, *Amaranthus* has been shown in recent investigations to possess greater digestibility of both dry matter and crude protein, supporting its role as a viable alternative source of dietary protein and fiber for ruminants. Its favorable fermentation characteristics, including high volatile fatty acid (VFA) concentrations, total gas production, and ammonia-N levels, suggest excellent feed quality [5]. Additionally, *Amaranth* has been shown to partially or fully replace soybean or fishmeal in poultry, fish, pig, and rabbit diets [6]. Moreover, Studies using in vitro and in situ methods demonstrate that *Amaranth* forages and grains have good to excellent digestibility and degradability for ruminants and other livestock [7]. Two *Amaranth* varieties (*Kharkovskiy* and *Sem*) have shown promising nutritional profiles, while diets containing up to 105 g/kg DM of *Amaranth* silage improved crude protein intake, dry matter intake, and milk yield in lactating Holstein cows [8,9]. Although *Amaranth* shows potential as a sustainable forage, the roles of its bioactive compounds, including flavonols, hydroxycinnamic acids, and benzoic acids, in ruminant nutrition remain unclear. To better understand their influence on bovine health, future studies should focus on isolating the functional components and uncovering their mechanisms of action.

Maintaining mammary gland health and regulating inflammation are essential for lactating dairy cows, as excessive inflammatory responses can impair milk production, disrupt metabolic homeostasis, and increase the risk of diseases such as mastitis. While inflammation is a critical immune defense mechanism, uncontrolled activation leads to oxidative stress and tissue damage, necessitating effective regulation [10,11]. *Amaranth* has shown potential as a valuable feed ingredient in dairy production due to its rich nutritional profile, including high lysine content that complements conventional cereal-based diets and a unique lipid fraction containing squalene, tocopherols, and sterols, which contribute to antioxidant and hypocholesterolemic effects [12]. Additionally, *Amaranth* exhibits anti-inflammatory properties, with recent studies identifying bioactive peptides from germinated *Amaranth* that modulate inflammatory pathways. Glutamine and poly-glutamine contained within these peptides are likely to mediate anti-inflammatory responses by inhibiting activation of the NF-κB pathway [13]. However, despite reports of its anti-inflammatory potential [14], the specific bioactive compounds and underlying mechanisms remain unclear.

Network pharmacology offers a systematic approach to identifying active components and elucidating their multi-target effects, aligning with the complex pharmacological mechanisms of traditional Chinese medicine [15,16]. By integrating network analysis with molecular docking, which predicts ligand-receptor interactions [17], this approach facilitates the precise selection of functional compounds and the exploration of their mechanisms of action. Molecular Dynamics Simulation(MDS) has become a powerful approach for analyzing critical structural features at protein–ligand interfaces, including intermolecular hydrogen bonding, contact surface area, and binding energy, thereby serving as an essential tool for elucidating biomolecular functions and accelerating drug discovery. These simulations offer valuable insights into ligand–receptor interactions and can significantly streamline the drug development pipeline. Consequently, further investigations are warranted to elucidate the bioactive properties of *Amaranth*, optimize its dietary incorporation, and assess its effects on mammary gland function and lactation performance. The application of such advanced analytical methods may provide a robust scientific basis for promoting the use of *Amaranth* in dairy cow nutrition and health management.

A combination of network pharmacology, docking analysis, and molecular dynamics simulation was employed to elucidate the bioactive components, relevant targets, and anti-inflammatory mechanisms associated with *Amaranth*. By integrating computational and experimental strategies, we aim to uncover the multi-target interactions of *Amaranth* in inflammatory pathways, contributing to a deeper understanding of its potential benefits for mammary gland health. This study not only addresses the existing knowledge gap but also supports the development of sustainable dietary interventions to enhance lactation performance in dairy cattle, aligning with the need for environmentally friendly feed alternatives in modern dairy production.

## Materials and methods

*Amaranth* powders were collected at four distinct growth stages, including the bud stage (XL), early flowering stage (CH), full flowering stage (SH), and maturity stage (CS). All samples were obtained from the Hubei Key Laboratory of Animal Nutrition and Feed Science, Wuhan Polytechnic University, Wuhan, China. Chemical composition was determined using established methodologies. Specifically, crude protein (CP) was derived from nitrogen content using the AOAC 2001.11 conversion factor (N × 6.25). Ether extract (EE) and ash content were assessed in accordance with AOAC 2003.05 and 942.05, respectively. The levels of NDF and ADF were evaluated following the technique described by Goering and Van Soest. Salicylic acid and selenium were quantified using standard protocols, calcium (Ca) by atomic absorption spectrophotometry, and phosphorus (P) by spectrophotometry.

### LC-MS/MS analysis of *Amaranth*

A sample of 100 mg *Amaranthus* powder was extracted using 500 μL of a methanol–water solution (4:1, v/v) containing an internal standard at a concentration of 10 μg/mL. The extraction process involved vortexing for 30 seconds, followed by homogenization at 45 Hz for 4 minutes. Subsequently, the mixture underwent ultrasonic treatment in an ice-water bath for 1 hour. After this, it was incubated at −40 °C for 60 minutes and then centrifuged at 12,000 rpm (equivalent to 13,800 × g, radius 8.6 cm) for 15 minutes at 4 °C. The resulting supernatant was passed through a 0.22 μm filter and transferred into vials for UHPLC-QE-MS analysis. A quality control (QC) sample was generated by combining 60 μL aliquots from each extract.

Chromatographic separation was conducted using a Vanquish UHPLC system (Thermo Fisher Scientific) equipped with a Waters UPLC BEH C18 column (1.7 μm, 2.1 mm × 100 mm). The mobile phases consisted of solvent A (0.1% formic acid in water) and solvent B (0.1% formic acid in acetonitrile), delivered at a flow rate of 0.4 mL/min. The gradient elution was programmed as follows: 0–3.5 min, A from 95% to 85%; 3.5–6 min, A from 85% to 70%; 6–6.5 min, A maintained at 70%; 6.5–12 min, A from 70% to 30%; 12–12.5 min, A maintained at 30%; 12.5–18 min, A from 30% to 0%; 18–25 min, A maintained at 0%; 25–26 min, A increased from 0% to 95%; 26–30 min, A maintained at 95%. An Orbitrap Exploris 120 mass spectrometer (Thermo Fisher Scientific) operated in FullScan-ddMS² mode under the control of Xcalibur software was employed for mass spectrometric analysis. The parameters were set as follows: sheath gas flow rate, 30 Arb; auxiliary gas flow rate, 10 Arb; ion transfer tube temperature, 350°C; vaporizer temperature, 350°C; primary MS resolution, 60,000; secondary MS resolution, 15,000; collision energy, 16/38/42 (NCE mode); and spray voltage, + 5.5 kV (positive mode) and −4.0 kV (negative mode) [18]. All analyses were conducted by Shanghai Biotree Biotech Co Ltd. A pooled quality control (QC) sample was prepared by combining 60 μL aliquots from each extract and injected every eight samples to monitor instrument stability. The relative standard deviation (RSD) of peak areas for internal standards and representative metabolites in QC samples was below 15%, confirming high technical reproducibility. All samples from the four growth stages were analyzed in triplicate (n = 3). Metabolite identification was accepted when the mass error was < 5 ppm, MS/MS spectral similarity exceeded 80% (against in-house and public databases), and retention time variation across replicates was within 0.5 min. Quantification was based on peak area normalization to the internal standard, with intra-group RSD < 20% for over 95% of the 266 identified metabolites. These criteria collectively ensured the robustness and reproducibility of the metabolomic data.

### Potential targets of *Amaranth* against inflammation

A total of 266 components were identified in *Amaranth* through metabolomic analysis, all of which were subsequently utilized for target prediction via network pharmacology. To predict the potential targets associated with *Amaranth*, compound-related data were collected from PubChem and SwissTargetPrediction databases. In parallel, genes related to inflammatory processes were obtained via the GeneCards platform. To maintain annotation consistency across datasets, all protein targets were unified and normalized using the UniProt database. By intersecting *Amaranth*-derived targets with inflammation-associated genes, a shared set of 321 overlapping targets was identified. This intersection was graphically represented using Venny 2.1.

### Target interaction network and hub gene extraction

To construct the protein-protein interaction (PPI) network, the shared targets were analyzed using STRING (v12.0), employing *Bos taurus* as the model species and applying a high-confidence interaction threshold of 0.9. The resulting interaction data were further analyzed in Cytoscape (Version 3.9.1) for visualization and network topology assessment. The CytoNCA tool was employed to evaluate node importance, and targets showing DC values more than twofold above the median were selected as core targets [19].

### Gene ontology (GO) and Kyoto Encyclopedia of Genes and Genomes (KEGG) enrichment analysis

The DAVID database was utilized to perform GO and KEGG enrichment analyses, aiming to uncover the biological processes and signaling cascades linked to the key targets implicated in the anti-inflammatory effects of *Amaranth*. The official gene symbol was used as the selected identifier, and the species was set to *Bos taurus*. Enrichment terms meeting the statistical criterion of *P* < 0.01 were included, and their visualization was performed via the Bioinformatics online tool (https://www.bioinformatics.com.cn/).

### *Amaranth* treatment of inflammation core target screening and network construction

To pinpoint the principal signaling cascades involved in *Amaranth*-mediated inflammation regulation, all inflammation-associated genes were first retrieved from the DAVID database and then intersected with *Amaranth* core targets based on KEGG pathway annotations. A comprehensive “*Amaranth*–inflammation–key targets–signaling pathways” network was then constructed using Cytoscape 3.9.1. The core target interactions were examined through STRING-based PPI analysis, with network visualization carried out using Cytoscape 3.9.1.

### Molecular docking analysis

To investigate the binding interactions between *Amaranthus*-derived compounds and inflammation-associated targets, molecular docking was employed. Protein crystal structures were downloaded from the PDB (https://www.rcsb.org/), and corresponding ligands were obtained via the PubChem database (https://pubchem.ncbi.nlm.nih.gov/). Protein preparation involved the removal of small molecules and water using PyMOL (https://pymol.org/). Hydrogen atoms were added using AutoDock Tools (Version 1.5.6, Scripps Research, USA), and the ligands’ rotatable bonds were identified and defined. The docking process utilized AutoDock Vina [20], with active site validation based on the positioning of small molecular ligands within the receptor. The docking grid box was centered on the receptor’s active site, defined by the position of the co-crystallized ligand, with dimensions of 22 × 24 × 22 Å (x, y, z) to fully encompass the binding pocket. The exhaustiveness parameter was set to 32 (four times the default) to enhance sampling accuracy. Reliability was assessed by redocking the native ligand from each PDB structure, which produced RMSD values below 2.0 Å in all cases, confirming the robustness of the docking protocol. Only docking poses with binding energies ≤ −4.0 kcal/mol and consistent interaction patterns—such as hydrogen bonding and hydrophobic contacts—were retained for further analysis. The docking box was set according to the receptor’s active site, ensuring accurate ligand-receptor interactions. The docking results were visualized using PyMOL and Discovery Studio 2019 (http://www.discoverystudio.net/) [21].

### Molecular dynamics simulation

The dynamic properties and binding robustness of protein–ligand complexes were further evaluated through molecular dynamics simulation implemented in GROMACS 2022. To replicate a realistic simulation environment, the system was maintained at steady temperature and pressure conditions, employing periodic boundary conditions. The protein was parameterized using the AMBER14SB force field, a standard and widely validated choice for protein simulations, and the ligands were parameterized using the General Amber Force Field (GAFF). Hydrogens were incorporated through the TIP3P solvation model, and charge neutrality was achieved by introducing Na⁺ counterions. The system then underwent minimization of potential energy, followed by controlled thermal equilibration from 0 K to 300 K over a 30 ps timescale. Equilibration was performed first for 100 ps under an NVT ensemble to stabilize temperature, followed by 1 ns under an NPT ensemble to ensure proper system density and pressure. After equilibration, a 100 ns MDS simulation was conducted at 298–300 K and 1 bar using periodic boundary conditions. Hydrogen bonding constraints were applied via the LINCS algorithm, employing a 2 femtosecond integration timestep. Electrostatic interactions at long range were computed using the Particle-Mesh Ewald (PME) approach with a cutoff of 1.2 nm, while non-bonded forces were limited to a 10 Å range. Trajectories were saved every 10 ps. RMSD analysis was performed to assess system stability, and results were visualized using Xmgrace and VMDS. Binding free energies were calculated using both MM/GBSA and MM/PBSA methods. Post-simulation, the resulting trajectories were analyzed using Visual Molecular Dynamics (VMDS) and PyMOL software. Binding free energy calculations were performed using the g_mmpbsa tool based on the Molecular Mechanics/Poisson-Boltzmann Surface Area (MM/PBSA) method.

### Statistical analysis

Data analysis was performed using SPSS 27 software. Following a significant one-way ANOVA, Duncan’s multiple range test was used as the post-hoc procedure to perform pairwise comparisons among group means and to identify which growth stages differed significantly. Data are expressed as mean ± standard deviation, and differences among groups were considered significant at P < 0.05 when marked with different lowercase letters.

## Results

### Nutritional composition dynamics across growth stages

No significant difference in dry matter content was observed between the bud stage (XL) and early flowering stage (CH) (*P* > 0.05); however, both were significantly lower than those at the full flowering (SH) and maturity (CS) stages (*P* < 0.05), suggesting enhanced biomass accumulation in the later stages of development. Crude protein content declined markedly as the plant matured (*P* < 0.05), while crude fat content peaked at the CH stage (*P* < 0.05). The highest crude ash content was recorded at the XL stage (*P* < 0.05). Both neutral detergent fiber (NDF) and acid detergent fiber (ADF) levels increased significantly with plant growth (*P* < 0.05). Calcium and phosphorus contents were highest at the CH stage, with significant variation among growth stages (*P* < 0.05) (Table 1). These results indicate substantial changes in the nutritional composition of *Amaranth* across developmental stages, providing a scientific basis for determining optimal harvest time, customizing feed formulations, and deepening the understanding of its physiological development.

**Table 1:** Nutritional components of dried *Amaranth* at different growth stages.

Nutritional components	CH	CS	XL	SH
DM (%)	91.35 ± 0.07^b^	91.61 ± 0.10^b^	93.37 ± 0.05^a^	93.99 ± 0.01^a^
CP (%)	19.99 ± 0.09^a^	16.16 ± 0.04^b^	15.02 ± 0.02^c^	13.98 ± 0.01^d^
EE (%)	8.32 ± 0.03^b^	8.62 ± 0.11^a^	8.44 ± 0.06^ab^	8.27 ± 0.13^b^
NDF(%)	56.90 ± 0.08^d^	63.41 ± 0.37^c^	64.13 ± 0.05^b^	64.92 ± 0.02^a^
ADF(%)	29.56 ± 0.04^d^	36.97 ± 0.05^c^	43.62 ± 0.11^b^	51.80 ± 0.14^a^
Ash (%)	18.01 ± 0.02^a^	15.30 ± 0.08^c^	15.15 ± 0.04^c^	16.59 ± 0.44^b^
Ca (%)	2.97 ± 0.05^ab^	3.45 ± 0.43^a^	3.06 ± 0.04^ab^	2.49 ± 0.07^b^
P (%)	16.68 ± 0.19^c^	19.24 ± 0.13^a^	17.53 ± 0.56^b^	16.05 ± 0.04^c^

### Qualitative analysis of *Amaranth*

**Fig 1:**
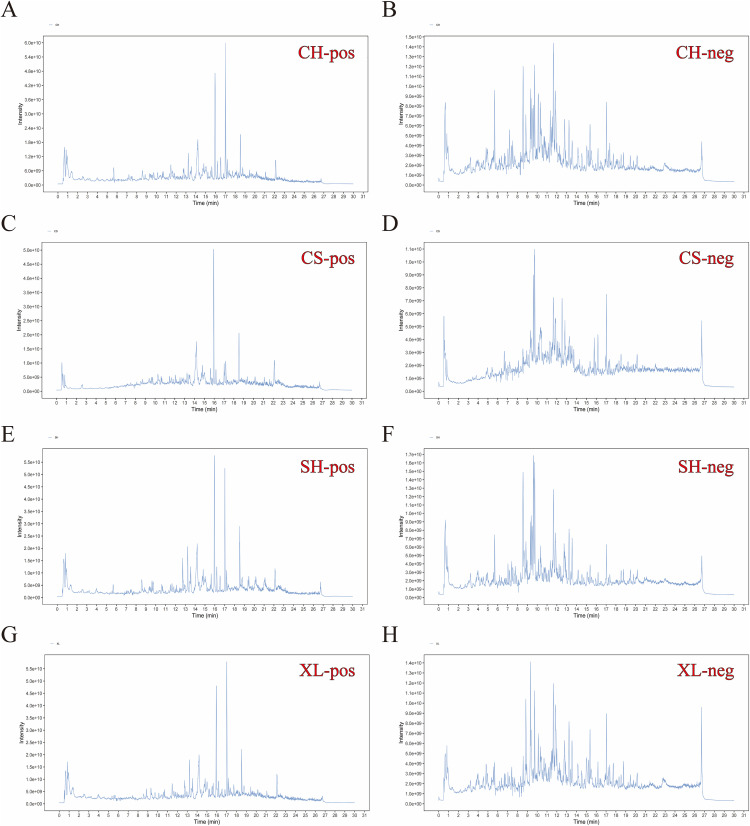
Qualitative analysis of *Amaranth* was conducted using UHPLC-MS/MS, enabling the structural identification of 20 compounds. These compounds included the top 10 detected in both positive and negative ion modes across four different growth stages: CH (early flowering stage, A–B), CS (maturity stage, C–D), XL (bud stage, E–F), and SH (full flowering stage, G–H). Each stage includes compounds identified in both negative and positive ion modes.

Qualitative analysis of *Amaranth* was conducted using LC-MS/MS, yielding comprehensive chromatographic profiles. Most compounds were detected within a retention time of 1–30 minutes. Structural identification was based on the m/z values and retention times of protonated molecular ions observed in each chromatographic peak (Fig 1), resulting in the identification of 20 representative compounds, including the top 10 detected in both positive and negative ion modes, as listed in Tables 2 and 3. In total, 266 active components were identified, encompassing phenols, alkaloids, polysaccharides, and glycosides. Notable constituents included 2-aminophenol, DL-3-phenyllactic acid, murracarpin, 12-hydroxyjasmonic acid, 3-p-coumaroylquinic acid, enoxolone, ferulic acid, isoferulic acid, sinapic acid, and 13-HODE. These findings suggest that the bioactivity of *Amaranth* is primarily attributed to its abundant phenolic acids, alkaloids, polysaccharides, and glycosides, providing a scientific basis for further exploration of its pharmacological potential.

**Table 2:** Information of the negative ion compounds from *Amaranth* tentatively characterized by LC-MS/MS.

PubChem CID	NameEN	Formula	2D Structure	mzmed	rtmed	MS2
14059029	(E)-5-((1R,3R,6S)-2,3-Dimethyltricyclo[2.2.1.02,6]heptan-3-yl)-2-methylpent-2-enoic acid	C_15_H_22_O_2_		233.1540276	762.916	M233.154T762.916
23815370	(1R,2R,4aS,6aS,6bR,9R,10R,11R,12aR)-1,10,11-trihydroxy-9-(hydroxymethyl)-1,2,6a,6b,9,12a-hexamethyl-2,3,4,5,6,6a,7,8,8a,10,11,12,13,14b-tetradecahydropicene-4a-carboxylic acid	C_36_H_58_O_10_		649.393567	845.155	M649.394T845.155
386476643	12:4 + 3O fatty acyl hexoside	C_18_H_28_O_9_		387.1660981	845.155	M649.394T845.155
5801	2-Aminophenol	C_6_H_7_NO		108.0453075	113.585	M387.166T298.251
3848	DL-3-Phenyllactic acid	C_9_H_10_O_3_		165.0552928	312.869	M165.055T312.869
483513	4-(2,3-Dihydroxy-3-Methylbutoxy)Furo(3,2-G)Chromen-7-One	C_16_H_16_O_6_		303.090406	328.538	M303.090T328.538
5319464	Murracarpin	C_16_H_18_O_5_		289.1107742	404.912	M289.111T404.912
72277	Epigallocatechin	C_15_H_14_O_7_		305.0689931	237.2315	M305.069T237.232
442456	Poncirin	C_28_H_34_O_14_		593.1861285	396.466	M593.186T396.466
5280863	Kaempferol	C_15_H_10_O_6_		285.0402087	512.267	M285.040T512.267

**Table 3:** Information of the positive ion compounds from *Amaranth* tentatively characterized by LC-MS/MS.

PubChem CID	NameEN	Formula	2D Structure	mzmed	rtmed	MS2
5497122	12-Hydroxyjasmonic acid	C_12_H_18_O_4_		227.1277227	833.092	M227.128T833.092
45359549	(1R,2R,4aS,6aS,6bR,9R,10R,11R,12aR)-1,10,11-trihydroxy-9-(hydroxymethyl)-1,2,6a,6b,9,12a-hexamethyl-2,3,4,5,6,6a,7,8,8a,10,11,12,13,14b-tetradecahydropicene-4a-carboxylic acid	C_30_H_48_O_6_		505.3518327	608.446	M505.352T608.446
9945785	3-p-Coumaroylquinic acid	C_16_H_18_O_8_		339.1077161	393.655	M339.108T393.655
5319332	[(1S)-5-hydroxy-1-[(2S,3R,4S,5S,6R)-3,4,5-trihydroxy-6-(hydroxymethyl)oxan-2-yl]oxy-1,4a,5,7a-tetrahydrocyclopenta[c]pyran-7-yl]methyl benzoate	C_22_H_26_O_10_		473.1433615	490.151	M473.143T490.151
10114	Enoxolone	C_30_H_46_O_4_		471.3475646	705.749	M471.348T705.749
5480982	Kaempferol 7-b-D-glucopyranoside	C_21_H_20_O_11_		449.1081172	355.325	M449.108T355.325
978	4-Aminobenzoic Acid	C_7_H_6_NO_2_		138.0547785	39.7232	M138.055T39.723
5319502	6,7-Dihydroxy-4-methylcoumarin	C_10_H_8_O_4_		193.0493236	1674.61	M193.049T1674.610
7410	Acetophenone	C_8_H_8_O		121.0647555	746.702	M121.065T746.702
190	Adenine	C_5_H_5_N_5_		136.0617096	49.8089	M136.062T49.809

### Acquisition of *Amaranth* and inflammation related targets

**Fig 2:**
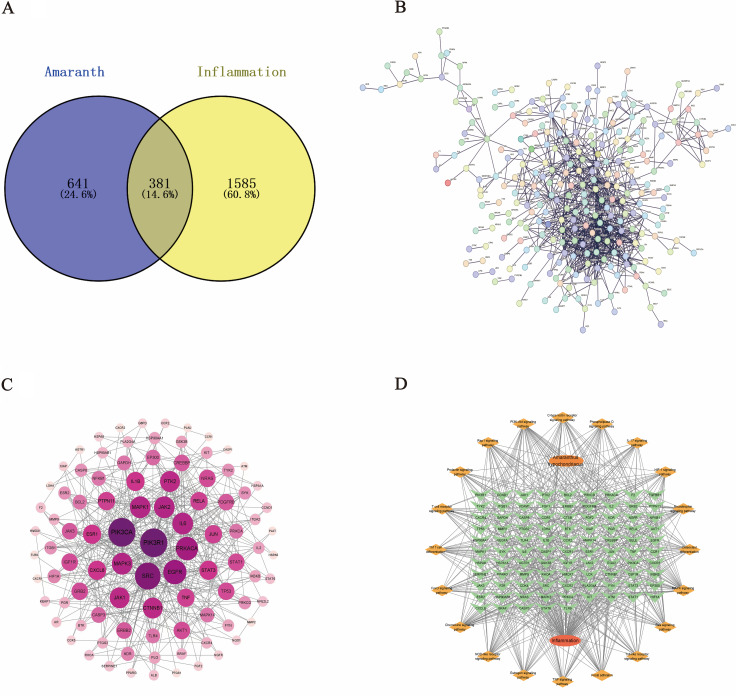
Network Pharmacology Analysis of *Amaranth* in Inflammation. (A) Venn diagram of common targets between *Amaranth* and inflammation; (B) PPI network construction and analysis using STRING; (C) PPI network of potential targets, with node color representing degree values (darker colors indicate higher degree values); (D)Interaction network of “drug-disease-key target-KEGG signaling pathway”.

Metabolomic profiling of *Amaranth* identified 266 compounds, which were subsequently subjected to target prediction using the PubChem and SwissTargetPrediction databases, yielding 1,022 potential targets. In parallel, 1,966 inflammation-related targets were obtained from the GeneCards database using “inflammation” as the keyword, with genes above the median relevance score retained. A Venn diagram analysis identified 381 overlapping targets between *Amaranth*-derived compounds and inflammation-related genes, as shown in Fig 2A.

### PPI network establishment and functional mapping of candidate targets

To clarify the potential anti-inflammatory mechanisms of *Amaranthus*, a total of 381 intersected targets were submitted to the STRING database for protein–protein interaction (PPI) analysis. The network was generated using a stringent confidence score cutoff (combined score > 0.9) to ensure high-quality interaction data. The resulting network contained 373 nodes and 842 edges (Fig 2B). Topological analysis based on degree, betweenness, and closeness centrality identified 132 key targets exceeding twice the median value. The core PPI network was visualized using Cytoscape (Fig 2C). Subsequently, the CytoHubba MCC algorithm was applied to rank the top eleven hub genes, including *GAPDH, ALB, IL6, TNF, IL1B, CASP3, STAT3, BCL2, MMP9,* and *EGFR*. The compound–disease–target–pathway network further highlighted the PI3K-Akt signaling pathway as a central regulatory axis (Fig 2D).

### Enrichment analysis of targets

**Fig 3:**
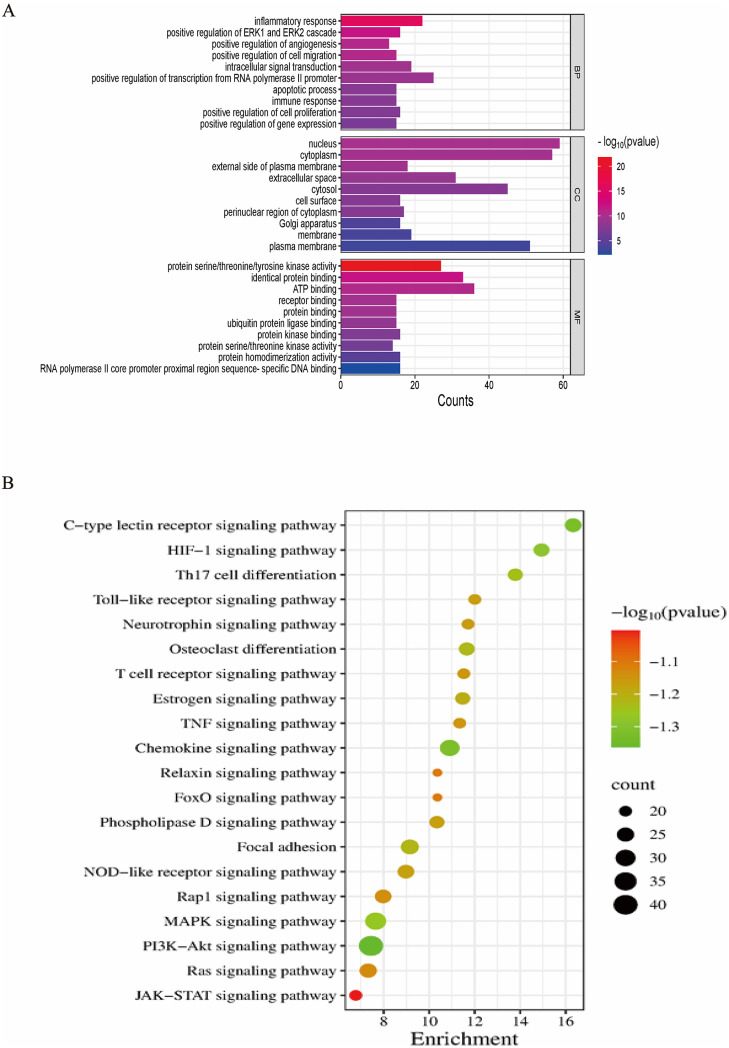
Enrichment results of GO and KEGG analysis. (A) GO functional enrichment analysis of the targets of *Amaranth* in the treatment of inflammation (top 10). (B) The bubble diagram of KEGG enrichment pathways (top 20).

The 132 core targets were subjected to Gene Ontology (GO) and Kyoto Encyclopedia of Genes and Genomes (KEGG) enrichment analyses using the DAVID database. GO terms were classified into biological processes (BP), cellular components (CC), and molecular functions (MF), ranked by log P-value, while KEGG pathways were prioritized based on target count (count > 15), statistical significance (P < 0.01), and fold enrichment (> 5). Network visualization (Fig 3) was based on the highest-ranking 10 GO terms and KEGG pathways identified through enrichment analysis. GO enrichment identified 238 terms related to BP, 36 to CC, and 62 to MF. As illustrated in Fig 3A, the core targets were predominantly associated with RNA polymerase II promoter-mediated transcriptional regulation, ATP binding activity, inflammatory processes, homotypic protein interactions, and localization within both the nucleus and cytoplasm. In the bar chart, the horizontal axis represents the proportion of genes, with bar length indicating gene count and color intensity reflecting statistical significance (red indicating lower P-values). KEGG analysis identified 85 enriched pathways, with the top 20 visualized in Fig 3B. PI3K-Akt signaling emerged as the most prominently enriched pathway, closely followed by the MAPK signaling, chemokine signaling, and focal adhesion pathways, indicating that these may be the key mechanisms underlying the anti-inflammatory effects of *Amaranth*.

### Molecular docking

**Fig 4:**
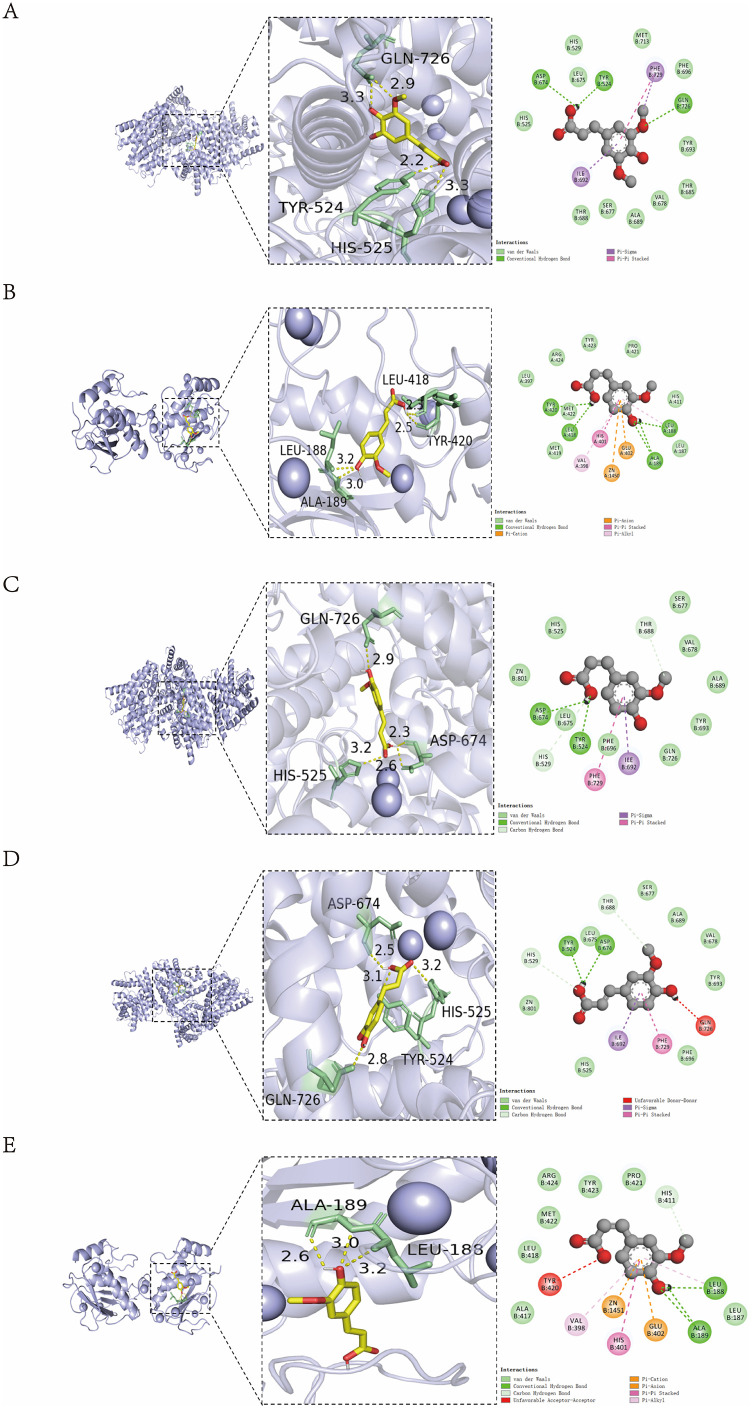
The molecular docking model of core anti-inflammatory targets of *Amaranth* with the core components of *Amaranth* and two dimensional patterns of bond (binding energy<− 7.5 kcal/mol). (A) *IL6* and Sinapic acid; (B) *MMP9* and Ferulic Acid; (C) *IL6* and Ferulic Acid; (D) *IL6* and isoferulic acid; (E) *MMP9* and isoferulic acid.

Eleven key targets—*GAPDH, ALB, IL6, TNF, IL1B, CASP3, STAT3, EGFR, BCL2, MMP9,* and *CTNNB1*—were selected based on pathway interaction analysis for molecular docking with four representative active compounds: ferulic acid, isoferulic acid, sinapic acid, and 13-HODE (Table 4). Strong binding was characterized by affinity values equal to or less than –4.0 kcal/mol, and increasingly negative scores were interpreted as markers of higher ligand–receptor stability. Among all docking results, the top five protein–ligand pairs with the highest affinities were *IL6*–sinapic acid, *IL6*–isoferulic acid, *MMP9*–ferulic acid, *IL6*–ferulic acid, and *MMP9*–isoferulic acid. Docking conformations with the lowest binding energies were visualized using PyMOL (Fig 4). The results revealed that sinapic acid and ferulic acid formed stable complexes with *IL6* and *MMP9* via multiple hydrogen bonds and electrostatic interactions. These structural insights provide a mechanistic understanding of the molecular interactions between *Amaranth*-derived compounds and inflammation-related targets, reinforcing the anti-inflammatory potential of *Amaranth* at the molecular level.

**Table 4:** Results of molecular docking between the main active components and key targets of *Amaranthus.*

Key target	Afinity/kcal·mol-¹			
	FeruliAcid	Isoferulicacid	Sinapicacid	13-HODE
*GAPDH*	−6.5	−6.5	−6.6	−5.6
*ALB*	−6.3	−6.2	−6.4	−5.5
*IL6*	−7.2	−7.3	−7.5	−5.9
*TNF*	−6.3	−6	−5.8	−5.6
*IL1B*	−5.5	−5.5	−5.4	−4.9
*CASP3*	−5.2	−5.2	−5.2	−4.4
*STAT3*	−5.9	−5.6	−5.6	−5.1
*EGFR*	−6.7	−6.5	−6	−5.4
*BCL2*	−6.2	−6.4	−5.7	−5.6
*MMP9*	−7.3	−7.1	−6.5	−5.9
*CTNNB1*	−5.6	−5.3	−5.3	−4.6

### Molecular dynamics simulation

**Fig 5:**
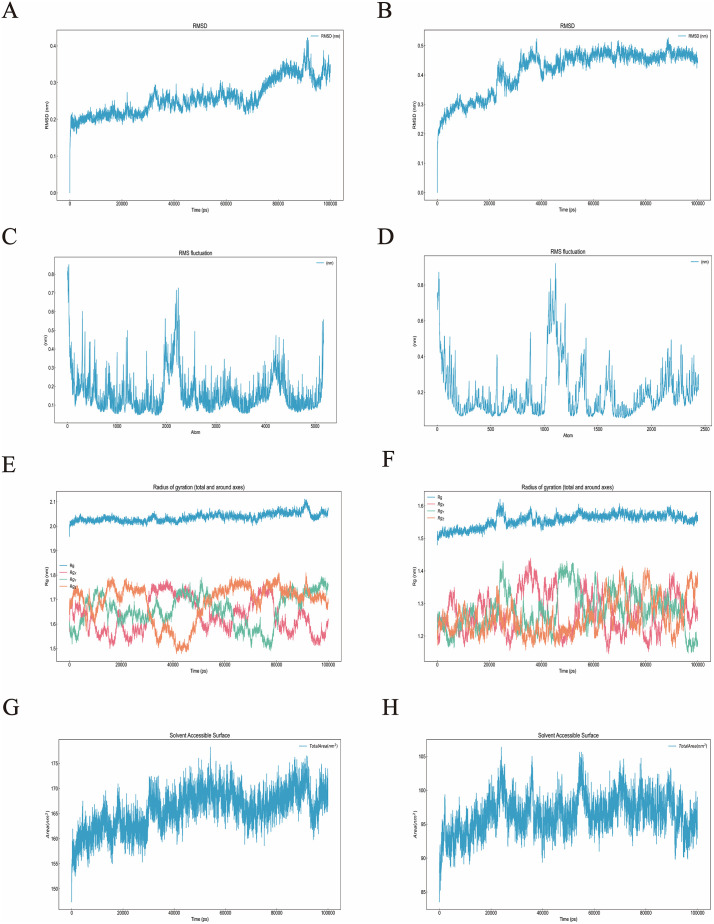
MDS simulation results of proteins *IL6*-Sinapic acid complex and *MMP9*-Ferulic Acid complex. (A) RMSD deviation of proteins *IL6* in complex with Sinapic acid; (B) RMSF deviation of proteins *IL6* in complex with Sinapic acid; (C) Rg of proteins *IL6* in complex with Sinapic acid; (D) SASA of proteins *IL6* in complex with Sinapic acid; (E) RMSD deviation of proteins *MMP9* in complex with Ferulic Acid; (F) RMSF deviation of proteins *MMP9* in complex with Ferulic Acid; (G) Rg of proteins *MMP9* in complex with Ferulic Acid; (F) SASA of proteins *MMP9* in complex with Ferulic Acid.

Based on the molecular docking outcomes, molecular dynamics simulations (MDS) were conducted to gain deeper insights into the stability and interaction dynamics of *Amaranth*-derived compounds with inflammation-associated protein targets. Two protein–ligand complexes with the lowest binding energies–*IL6*–sinapic acid and *MMP9*–ferulic acid–were selected for 100 ns MDS simulations. Conformational stability and interaction patterns of the complexes were elucidated through molecular dynamics simulations, wherein RMSD values were calculated to evaluate temporal structural fluctuations. As shown in Fig 5A and 5B, both complexes exhibited minimal RMSD fluctuations, remaining within 1 nm throughout the simulation, indicating stable binding conformations. The root mean square fluctuation (RMSF), which reflects the flexibility of individual amino acid residues, remained consistently low for both complexes (Fig 5C–D), suggesting that ligand binding had minimal impact on protein backbone dynamics. The radius of gyration (Rg), an indicator of structural compactness, showed stable trends with fluctuations within 0.07 nm (Fig 5E–F), further supporting the structural integrity of the complexes. Additionally, solvent-accessible surface area (SASA) values remained consistent throughout the simulation (Fig 5G–H), aligning with the RMSD and RMSF results. Collectively, these findings confirm that *IL6*–sinapic acid and *MMP9*–ferulic acid complexes maintain high conformational stability and tight binding throughout the simulation period, supporting their potential as stable anti-inflammatory interactions at the molecular level.

## Discussion

*Amaranth*, a high-yielding and climate-resilient crop, has emerged as a promising candidate in the pursuit of sustainable feed strategies for modern livestock systems. Its adaptability to marginal environments, along with a nutrient-rich profile comprising high-quality protein, essential fatty acids, flavonoids, and phenolic acids, positions it as a dual-purpose feed ingredient capable of supporting both animal performance and environmental goals. Recent attention has shifted toward its biofunctional attributes, particularly antioxidant and anti-inflammatory properties, which may contribute to immune modulation in livestock. While preliminary studies have suggested such potential, a detailed mechanistic understanding remains limited.

Mastitis, a prevalent and economically detrimental inflammatory disease in dairy cattle, impairs milk production and compromises animal health and welfare [22]. Although antibiotics remain the cornerstone of treatment, escalating concerns over antimicrobial resistance, drug residues, and regulatory constraints have intensified the demand for alternative strategies that enhance innate immunity and mitigate inflammation at its source. Against this backdrop, *Amaranth* has gained interest not only for its nutritional contributions but also for its potential role in modulating inflammatory processes in ruminants [23]. However, the specific compounds responsible for these effects, and their molecular mechanisms of action, remain largely undefined.

To explore these mechanisms, this study employed an integrative approach combining LC-MS/MS-based metabolomic profiling of *Amaranth* at different growth stages with network pharmacology, molecular docking, and molecular dynamics simulations. Among 266 identified metabolites, four were prioritized as key anti-inflammatory candidates: ferulic acid, isoferulic acid, sinapic acid, and 13-HODE. These compounds exhibited strong predicted affinities toward inflammation-associated targets such as *IL6* and *MMP9*. Enrichment analyses further revealed the involvement of PI3K-Akt and MAPK signaling pathways. Taken together, these results provide mechanistic insights into how *Amaranth* may influence immune regulation and inflammation, particularly in diseases such as bovine mastitis.

To contextualize the potential of *Amaranthus hypochondriacus* in livestock nutrition, we compare it with other traditional medicinal plants. Although *Curcuma longa* provides potent bioactive constituents, the complexity and cost of extraction and standardization limit its scalability in feed applications [24]. Similarly, *Panax ginseng* contains ginsenosides with anti-inflammatory activity but typically requires multi-step solvent extraction and a 4–6-year cultivation/harvest cycle, rendering routine feed inclusion economically impractical [25]. By contrast, *Amaranth* combines substantive nutritional value and anti-inflammatory efficacy with minimal processing (e.g., drying or ensiling), making it particularly suitable for sustainable, scalable biofunctional feed development.

### Bioactive compounds and the multi-target anti-inflammatory mechanism of *Amaranth*

Phenolic acids and oxidized fatty acids emerged as the principal classes of bioactives contributing to *Amaranth* anti-inflammatory potential. LC-MS/MS analysis identified ferulic acid, isoferulic acid, sinapic acid, and 13-HODE as dominant constituents. Network pharmacology predictions indicated their involvement in multiple inflammation-related targets and pathways, suggesting a polypharmacological mode of action.

Ferulic acid (FA), a well-characterized phenolic compound, has been widely reported to exhibit both antioxidant and anti-inflammatory activities. It scavenges reactive oxygen species (ROS), modulates key antioxidant enzymes, and interferes with pro-inflammatory cascades such as PPAR-γ, NF-κB, and MAPK pathways Notably [26,27], FA has demonstrated the capacity to suppress apoptosis and inflammatory cytokine production in LPS-stimulated bovine mammary epithelial cells, underscoring its relevance to mastitis pathogenesis [28]. In line with these findings, our study confirmed FA’s strong binding affinity to *IL6* and *MMP9*, further validated through molecular dynamics simulations.

Isoferulic acid, a structural analog of FA, also showed robust interaction with inflammatory mediators. Previous studies have demonstrated that it can suppress the nuclear translocation of NF-κB and mitigate MAPK pathway activity by reducing the phosphorylation levels of p38 and ERK [29,30]. Its documented antioxidative and antiapoptotic effects in models of diabetic nephropathy and cancer broaden its therapeutic scope [31,32].In our analysis, isoferulic acid demonstrated comparable binding behavior to FA, reinforcing its potential role in inflammatory modulation.

Sinapic acid (SA) represents another potent phenolic acid detected in *Amaranth*. Its ability to selectively inhibit NLRP3 inflammasome activation—while sparing upstream priming events—positions it as a unique modulator of innate immunity [33]. Additional studies have highlighted its effectiveness in suppressing joint inflammation and regulating lipid metabolism through nanoparticle delivery systems. Molecular docking confirmed stable interactions between SA and *IL6*, implying its contribution to the plant’s overall immunomodulatory profile [34].

Although 13-HODE has been less extensively studied in the context of inflammation, its identification as a key metabolite suggests that oxidized linoleic acid derivatives in *Amaranth* may participate in lipid-mediated signaling pathways relevant to inflammation. Further functional validation is essential to elucidate its biological significance and therapeutic relevance.

Collectively, these findings indicate that *Amaranth* exerts anti-inflammatory effects through a combination of synergistic bioactives targeting multiple components of inflammatory signaling. This supports its application not only as a nutritive feed component but also as a phytogenic additive for improving immune health in dairy cattle.

### Key targets of *Amaranth* in inflammation modulation

To decipher the molecular interface between *Amaranth*-derived compounds and inflammation, network pharmacology was employed to identify core protein targets. *GAPDH*, *ALB*, and *IL6* emerged as central nodes in the protein–protein interaction network, revealing a complex web of immunometabolic regulation.

*GAPDH*, traditionally known for its glycolytic role, has gained recognition as an immune regulator. Post-translational modifications such as malonylation have been shown to influence its role in cytokine regulation, particularly in activated macrophages [35,36]. In bovine cells, *GAPDH* expression is sustained upon LPS challenge, indicative of its involvement in inflammation [37]. Additionally, secreted *GAPDH* can shift macrophage responses by downregulating *TNF-α* and enhancing *IL-10* expression [38]. These immunoregulatory functions align with our findings, suggesting *GAPDH* as a functional target for *Amaranth*-derived bioactives.

Albumin (*ALB*) also emerged as a key node. Beyond its role as a transport protein, *ALB* contributes to redox balance by scavenging reactive oxygen and nitrogen species [39,40]. Its interactions with membrane receptors such as FcRn and gp60 amplify its anti-inflammatory potential through extended circulation and molecular delivery. Targeting *ALB* may therefore provide systemic antioxidant benefits, reinforcing the hypothesis that *Amaranthus* influences immune function via oxidative stress pathways.

Interleukin-6 *(IL6*), a multifunctional cytokine with established roles in mastitis, was another high-confidence target. *IL6* levels are markedly elevated in inflamed mammary tissues, particularly during Streptococcus spp [41,42]. Infections Given its dual roles in acute-phase inflammation and resolution via trans- and classic signaling [43,44]. *IL6* represents a strategic node for immunomodulation. Molecular docking revealed strong affinities between *IL6* and multiple *Amaranth*-derived metabolites, supporting its role as a primary mediator of the observed anti-inflammatory effects.

These targets collectively underscore the capacity of *Amaranth* to modulate inflammation not through single-gene interaction, but via a coordinated network of molecular effectors.

### Key pathways of *Amaranth* in inflammation modulation

Functional enrichment of the identified targets revealed three major signaling axes: PI3K-Akt, MAPK, and chemokine pathways. These signaling cascades are central to the orchestration of inflammatory responses and are directly implicated in mastitis pathogenesis. Among them, the PI3K-Akt pathway serves as a pivotal regulator of immune homeostasis. It is activated in bovine mammary tissue by bacterial components such as lipoteichoic acid via the TLR2/MyD88/PI3K-Akt axis [45]. Variants within PI3K-Akt-related immune genes have been linked to mastitis susceptibility or resilience [46]. Our findings suggest that *Amaranth*-derived compounds may exert their effects by modulating this pathway, thereby offering therapeutic potential for mastitis mitigation. Ferulic acid has been reported to activate the PI3K–Akt pathway in models of barrier dysfunction and inflammation. In LPS-stimulated Caco-2 cells, ferulic acid upregulates miR-200c-3p, which suppresses PTEN, thereby enhancing PI3K–Akt signaling and improving epithelial barrier integrity [47]. Sinapic acid exerts anti-inflammatory effects by inhibiting NF-κB signaling, leading to reduced expression of pro-inflammatory cytokines (TNF-α, IL-6), mitigation of oxidative stress, and decreased immune-cell infiltration as indicated by MPO [48].

The MAPK pathway, activated notably by IL-17A, amplifies inflammatory cascades through NF-κB and cytokine release [49]. This was consistent with our KEGG results, and aligns with reports of miRNA-mediated suppression of MAPK and NF-κB signaling in bovine mammary cells [50]. Similarly, chemokine signaling, which coordinates leukocyte recruitment and retention, plays a central role in chronic inflammation characteristic of persistent mastitis [51].

These findings provide compelling evidence that *Amaranth* modulates inflammation through concurrent regulation of key immunological signaling circuits. However, translating these insights into clinical applications necessitates further confirmation through well-controlled in vivo trials.

### Molecular docking and dynamics simulations of *Amaranth* targets

The binding interactions between principal metabolites from *Amaranth* and inflammatory proteins were validated through a combination of molecular docking and dynamic simulation techniques. Ferulic acid and sinapic acid exhibited strong docking scores against *MMP9* and *IL6*, respectively. These findings were corroborated by 100 ns molecular dynamics simulations, which demonstrated stable RMSD, consistent SASA, and minimal atomic fluctuations, indicating stable complex formation. This computational validation strengthens the hypothesis that the anti-inflammatory activity of *Amaranthus* is mediated through specific, high-affinity interactions with key inflammatory proteins. While the in silico approach accelerates target identification and mechanism elucidation, experimental validation in biological systems remains indispensable. Further studies employing bovine mammary epithelial cells and mastitis models are warranted to confirm efficacy, assess bioavailability, and determine safety profiles. Optimization of formulation and dosing strategies will also be essential to facilitate practical implementation in dairy production systems.

In conclusion, this study highlights the multi-target anti-inflammatory potential of *Amaranthus* by employing an integrative strategy combining metabolomics, network pharmacology, molecular docking, and molecular dynamics simulations. Using LC-MS/MS, we identified 20 representative bioactive compounds—most notably ferulic acid, isoferulic acid, sinapic acid, and 13-HODE—which demonstrated strong predicted interactions with inflammation-associated proteins. Subsequent network-based analyses revealed that these compounds regulate key pathways involved in inflammation, including PI3K-Akt, MAPK, and chemokine signaling. GO and KEGG enrichment further supported these findings, while PPI network analysis identified *GAPDH*, *ALB*, and *IL6* as central molecular targets.

These results offer a mechanistic foundation for the use of *Amaranthus* as a functional feed additive, particularly in supporting mammary gland health and mitigating inflammatory disorders such as mastitis in dairy cattle. In addition to expanding the phytochemical database of *Amaranthus*, this research underscores its value in sustainable livestock nutrition. Future in vivo validation is necessary to confirm the biological efficacy and application potential of these findings in animal health management.

## Conclusions

This study applied LC-MS/MS technology to identify 20 key bioactive compounds in *Amaranth*, including ferulic acid, isoferulic acid, sinapic acid, and 13-HODE. Combined with network pharmacology analysis, these compounds were shown to exert anti-inflammatory activity by targeting multiple pathways. GO and KEGG enrichment analyses of 132 inflammation-related targets revealed that *Amaranth* primarily modulates PI3K-Akt, MAPK, and chemokine signaling, among others. Protein–protein interaction network analysis further identified *GAPDH, ALB*, and *IL6* as key regulatory nodes. These results provide a theoretical basis for the development and functional utilization of *Amaranth* as a natural anti-inflammatory resource. Moreover, the findings enrich the chemical composition database of *Amaranth* and support its potential application as a functional feed ingredient for managing inflammation in dairy cows. Further *in vivo* studies are warranted to verify these mechanisms and support practical implementation in animal health management.

## Supporting information

S1 Fig Supplementary Figure 1. (TIF)

S1 Table Software and Tool Versions. (DOCX)

S1 File List of 266 identified compounds from Amaranth. (XLSX)

S2 File List of 381 overlapping targets. (XLSX)

S3 File List of 132 Key targets. (XLSX)

S4 File GO enrichment results. (XLSX)

S5 File KEGG enrichment results. (XLSX)
