# Supplementary material for: A screening strategy for bioactive components from Amaranth: An integrated approach of network pharmacology, molecular docking and molecular dynamics simulation
Source: PLoS One. 2025 Dec 26;20(12):e0338443. doi: 10.1371/journal.pone.0338443 (PMC12742726; doi:10.1371/journal.pone.0338443)
Supplement: S1 Table — (DOCX) [file pone.0338443.s002.docx]

| Category | Tool/Database | Version | Purpose | Website/Reference |
| --- | --- | --- | --- | --- |
| Target Prediction | PubChem | 2023 release | Compound structure and target retrieval | https://pubchem.ncbi.nlm.nih.gov/ |
| Target Prediction | SwissTargetPrediction | 2021 update | Protein target prediction | http://www.swisstargetprediction.ch/ |
| Target Prediction | UniProt | 2024_04 | Protein ID normalization | https://www.uniprot.org/ |
| Inflammation Genes | GeneCards | v5.18 | Inflammation-related gene mining | https://www.genecards.org/ |
| Protein-Protein Interaction | STRING | v12.0 | PPI network construction (confidence > 0.9) | https://string-db.org/ |
| Network Visualization | Cytoscape | 3.9.1 | Network topology and visualization | https://cytoscape.org/ |
| GO/KEGG Enrichment | DAVID | 2021 | Functional enrichment (P < 0.01) | https://david.ncifcrf.gov/ |
| Visualization | Bioinformatics online tool | 2023 platform | GO/KEGG bar and bubble plots | https://www.bioinformatics.com.cn/ |
| Venn Diagram | Venny | 2.1 | Target intersection visualization | https://bioinfogp.cnb.csic.es/tools/venny/ |
| Molecular Docking | PDB (Protein Data Bank) | v2.5.0 | Protein preparation and visualization | https://pymol.org/ |
| Molecular Docking | AutoDock Tools | 1.5.6 | Protein and ligand preparation | [https://autodocksuite.scripps.edu/adt/](https://autodocksuite.scripps.edu/adt/) |
| Molecular Docking | Discovery Studio | 2019 | 2D interaction visualization | [http://www.discoverystudio.net/](http://www.discoverystudio.net/) |
| Molecular Dynamics | GROMACS | 2022 | Molecular dynamics simulation | http://www.gromacs.org/ |
